# Autoimmune diseases and gut microbiota: a bibliometric and visual analysis from 2004 to 2022

**DOI:** 10.1007/s10238-023-01028-x

**Published:** 2023-03-01

**Authors:** Youao Zhang, Yongzheng Peng, Xu Xia

**Affiliations:** 1 The First School of Clinical Medicine , Southern Medical University, Guangzhou, 501515 China; 2 Department of Transfusion Medicine and Department of Laboratory Medicine, Zhujiang Hospital of Southern Medical University, Guangzhou, 510282 China; 3 Southern Medical University Library, No.1023, South Shatai Road, Baiyun District, Guangzhou, 510515 Guangdong China

**Keywords:** Autoimmune diseases, Gut microbiota, Visual analysis, Hot spots, Frontiers, Keywords analysis

## Abstract

Many studies have shown that gut microbiota is closely related to autoimmune diseases (ADs). Studies on gut microbiota and ADs have also increased significantly, but no bibliometric analysis has summarized the association between gut microbiota and ADs. This study aimed to conduct a bibliometric and visual analysis of published studies on gut microbiota and ADs. Based on the Web of Science Core Collection SCI-expanded database, we utilize Excel 2019 and visualization analysis tools VOSviewer and co-occurrence13.2 (COOC13.2) for analysis. A total of 2516 related kinds of literature were included, and the number of papers presented an overall increasing trend. The country/region with the most publications is the USA, the institution is the Harvard Medical School, and the author is Mikael Knip from the USA. Hot research areas include intestinal regulation (such as dysbiosis, short chain fatty acids, and probiotics), multisystem ADs (such as multiple sclerosis, rheumatoid arthritis, and inflammatory bowel disease), and immune-related cells (such as T cells, and dendritic cells). Psoriasis, dysbiosis, autoimmune liver disease, and fecal microbiota transplantation may be the future research direction. Our research results can help researchers grasp the current status of ADs and gut microbiota research and find new research directions in the future.

## Introduction

In the past few decades, the prevalence of autoimmune diseases (ADs) has increased rapidly worldwide, affecting up to 10% of the population, and most human ADs are complex diseases caused by the interaction between genetic, epigenetic, and environmental factors [1–4]. ADs are also associated with psychological stress and gastrointestinal symptoms, namely microbiota dysregulation, intestinal hyperpermeability, and intestinal inflammation [2, 5, 6]. They are characterized by chronic, systemic, excessive immune activation and inflammation and involve almost all body tissues [7, 8]. Glucocorticoids, non-steroidal anti-inflammatory drugs (NSAIDs), immunosuppressants, and biologics are currently used to treat ADs of different origins [3, 9]. Several dietary and natural products (including polyphenols, quercetin) have also been investigated as possible alternative therapeutic strategies for managing ADs [3, 10]. However, due to the complexity of ADs, the social burden caused by ADs is still severe.

The role of the gut microbiome in human disease has received much attention, and the understanding of the composition and function of the gut microbiome has increased exponentially [11, 12]. Primarily responsible for maintaining the balance between host defense and immune tolerance, the gut microbiota plays a crucial role in shaping the immune system [11, 13, 14]. Dysbiosis of the gut microbiota is associated with various alterations in the immune system [11, 13]. The possible causal relationship between the gut microbiota and the initiation or exacerbation of ADs, microbial dysbiosis, and intestinal leakage are common phenomena in human ADs and mouse models of autoimmunity [11, 15]. Gut commensal microbiota can contribute to the pathogenesis of ADs by altering the intestinal barrier [16, 17]. Among them, the effects on gut microbiota through probiotics and fecal transplantation may serve as novel targets for autoimmune therapy [18].  It can be seen that gut microbiota plays a vital role in ADs. Therefore, we want to understand the research hot spots and future trends of ADs and gut microbiota more intuitively and comprehensively through bibliometric methods.

The bibliometric analysis uses mathematical and statistical methods to study the distribution, structure, quantity, and content evolution of bibliographic information qualitatively or quantitatively. It is of great value to describe the status quo of various research disciplines, publishing trends, and scientific achievements of researchers, institutions, and countries, as well as future research hot spots, academic frontiers, and knowledge maps, which provide researchers and clinicians a comprehensive picture of the current state of development in a particular research area [19, 20]. Moreover, bibliometrics has been widely used in immunology [19–25]. And VOSviewer is also a commonly used software in various fields of bibliometrics [21–30]. At the same time, COOC is a software developed by Chinese scholars for bibliometrics and scientific mapping and is continuously iterating [26]. COOC software has also been increasingly used in SCI-E articles [26–29]. It can be seen that bibliometric analysis is an excellent choice for the study of ADs and gut microbiota. Still, there need to be relevant studies on analyzing the whole literature by bibliometrics of ADs and gut microbiota. This paper aims to make up for the shortcomings of this study and summarize the studies of ADs and gut microbiota to some extent.

## Material and methods

### Data retrieval strategy, data extraction, and cleaning

The research object of this paper is the correlation study of ADs and gut microbiota. The web of science core collection is one of the most comprehensive and authoritative databases, containing more than 12,000 high-quality journals [19, 20, 31]. Thus, we select the web of science core collection SCI-expanded (SCI-E) database as the search source and as the data source of the research object and select the advanced search, the search formula: TS = (gut OR intestine OR gastrointestine OR gastrointestine) AND TS = (microbiota OR microbiome OR flora OR microflora OR bacteria) AND TS = (autoimmunity OR autoimmune). The time limit was from 2004-01-01 to 2022-12-20, and 2659 papers were retrieved, excluding duplicate publications, conference abstracts, letters, etc., mainly leaving articles and reviews. And without consulting in advance, we read the title, abstract, and keywords of the searched literature simultaneously, exclude irrelevant literature, and only include what we think can be left. In the end, a total of 2516 articles were left.

### Scientometric analysis methods

The 2516 pieces of literature were exported in plain text format. We utilize Excel 2019 and visualization analysis tools VOSviewer, co-occurrence13.2 (COOC13.2) for overall trend analysis, synonym merging, frequency of countries/regions, institutions, authors and funds, cluster analysis of co-occurrence matrix, dissimilarity matrix, two-mode matrix, burst keywords map to explore the research hot spot and frontier direction of ADs and gut microbiota.

## Results

### Annual analysis of publication

**Fig. 1:**
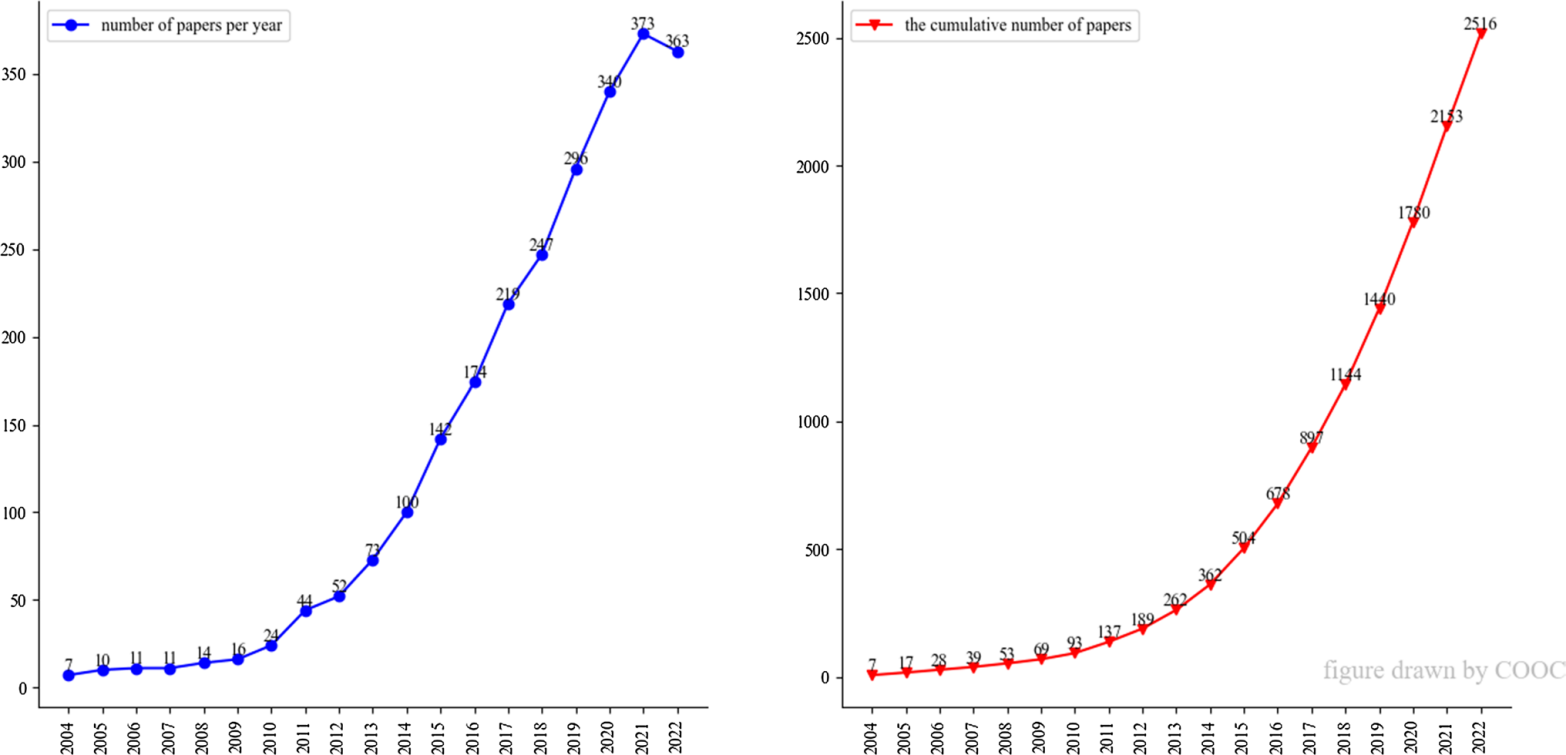
Number of papers per year and the cumulative number of papers. According to this figure, an uprising trend can be seen from 2012 to 2021, and the peak value appears in 2021

Since 2004, the research on ADs and gut microbiota has increased rapidly every year and only declined slightly in 2022 (Fig. 1). According to the increasing law of scientific literature, research in this direction is still rising.

### Country/region, institution, author, and journal frequency analysis

As seen from Table 1, the frequency analysis of countries/regions shows that the USA is the country with the most research on ADs and gut microbiota, and it is mainly concentrated in Europe and North America. In Asia and Oceania, only China, Japan, and Australia made the list. The most significant research institution is Harvard Medical School, with six from the USA and the other four from the University of Helsinki and the University of Turku in Finland, Karolinska Institute in Sweden, and the University of Milan in Italy. The most published authors are Mikael Knip from the University of Helsinki (23 articles). The second is Li Wen from Yale University. The third is F. Susan Wong from Cardiff University, and the fourth is Jorma Ilonen from the University of Turku. The fifth is Lloyd H. Kasper from the Geisel School of Medicine at Dartmouth College. Furthermore, the high-yield authors 6, 7, 8, and 10 were all from institutions in the USA. A total of seven high-yield authors were from US institutions. The top three journals were frontiers in immunology, international journal of molecular sciences, and frontiers in microbiology. The top 10 journals published a total of 574 articles. Meanwhile, three of the top 10 journals are immunology journals, three are microbiology journals, two are comprehensive journals, one is a biochemistry and molecular biology journal, and one is a nutrition journal. The types are not single, which proves to a certain extent that studies on ADs and gut microbiota involve multiple studies and have received attention from various fields.

**Table 1:** Top 10 countries/regions, institutions, authors and journals

Rank	Country/region	Count	Institution	Count	Author	Count	Journal	Count	2022 impact factor /JCR partition
1	USA	939	Harvard Med Sch (USA)	67	Mikael Knip	23	Frontiers in immunology	202	8.786/Q1
2	China	450	Yale Univ (USA)	48	Li Wen	22	International journal of molecular sciences	64	6.208/Q1
3	Italy	242	Univ Helsinki (Finland)	43	F. Susan Wong	21	Frontiers in microbiology	50	6.064/Q1
4	Germany	214	NYU (USA)	39	Jorma Ilonen	18	Nutrients	49	6.706/Q1
5	England	132	Univ Florida (USA)	39	Lloyd H. Kasper	18	PloS One	49	3.752/Q2
6	Australia	113	Univ Turku (Finland)	38	Luo Xin M	15	Scientific reports	42	4.996/Q2
7	Japan	112	Harvard Univ (USA)	35	Javier Ochoa Reparaz	15	Gut microbes	32	9.434/Q1
8	Canada	112	Baylor Coll Med (USA)	33	Jose U. Scher	13	Journal of autoimmunity	31	14.511/Q1
9	Spain	98	Karolinska Inst (Sweden)	31	Michael Maes	13	Microorganisms	28	4.926/Q2
10	Netherlands	92	Univ Milan (Italy)	30	Ramnik J. Xavier	13	Journal of immunology	27	5.426/Q2

### Authors, institutions, countries/regions, and analysis of cooperation

**Fig. 2:**
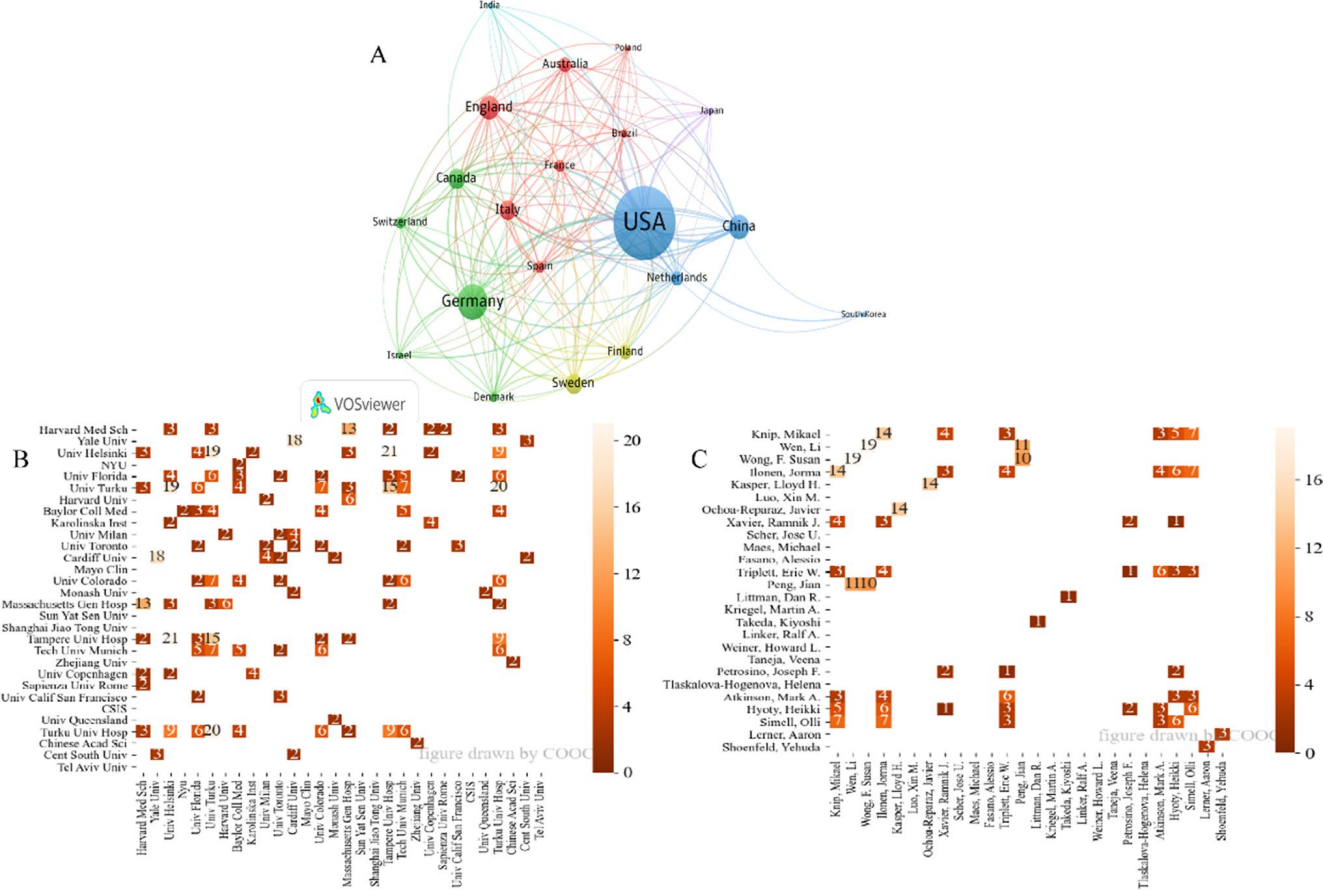
Countries/regions, institution, and author analysis. **A** Countries/regions cooperative network. **B** Institutions cooperative network. **C** Authors cluster map

It can be seen from Fig. 2A that the largest number of cooperation is between the USA and China. Meanwhile, the USA also has a close collaboration with Germany, Italy, England, and Canada. In regards to institutions, Fig. 2B, the University of Helsinki cooperated with Tampere University Hospital most times. The University of Turku and Tampere University Hospital, Yale University, and Cardiff University also collaborate closely. The cooperation between them is transnational. The University of Helsinki and the University of Turku also have a tight cooperative relationship. Their collaboration with each other is concentrated in the corresponding country. Furthermore, The University of Turku closely cooperates with Turku University Hospital, and Harvard Medical School cooperates closely with Massachusetts General Hospital. They are all part of the Turku and Harvard health care system, so it is not surprising that there is much collaboration. There are more transnational cooperation and cooperation between the same system on this topic. According to Fig. 2C, professors Li Wen and F. Susan Wong have cooperated most frequently. Mikael Knip and Jorma Ilonen, as well as Lloyd H. Kasper and Javier Ochoa-Reparaz, have the second most times of cooperation. Li Wen and F. Susan Wong also closely cooperate with Jian Peng. In general, the cooperation among authors is relatively concentrated.

### Citation analysis

According to Table 2, the most cited article was a 2009 review in nature reviews immunology by June L. Round and Sarkis K. Mazmanian, about the strong connection between the immune system and the gut microbiome. It is proposed that microbial disorders are associated with immune dysregulation (associated with autoimmunity) and that the immune system may be engineered or controlled by microbes [32]. This was followed by an article by Ivaylo I. Ivanov et al. in Cell in 2009. They found that colonization of the small intestine of mice with commensal segmented filamentous bacteria (SFB) was beneficial for the expression of genes related to inflammation and antimicrobial defense and increased resistance to the intestinal pathogen citrobacter rodentgens. Manipulating this symbiotic regulation may provide new opportunities for enhancing mucosal immunity and treating ADs [33]. The third most cited, but most frequently cited per year on average, was a review published in Cell in 2014 by authors Yasmine Belkaid and Timothy W. Hand. The dramatic rise in autoimmune and inflammatory diseases in some parts of the world, possibly coupled with the overuse of antibiotics, changes in diet, and elimination of nematodes, results in a lack of the resilient and diverse microbiota needed to build a balanced immune response, which plays a vital role in the induction, training, and function of the host immune system [34].

**Table 2:** Ranking of the top 10 highest cited references

Rank	Year	Title	Journal	First author	Citations
1	2009	The gut microbiota shapes intestinal immune responses during health and disease	Nature reviews immunology	June L. Round	3045
2	2009	Induction of intestinal Th17 cells by segmented filamentous bacteria	Cell	Ivaylo I. Ivanov	2965
3	2014	Role of the microbiota in immunity and inflammation	Cell	Yasmine Belkaid	2307
4	2008	Innate immunity and intestinal microbiota in the development of type 1 diabetes	Nature	Li Wen	1393
5	2013	Sex differences in the gut microbiome drive hormone-dependent regulation of autoimmunity	Science	Janet G. M. Markle	1145
6	2010	Gut-residing segmented filamentous bacteria drive autoimmune arthritis via T helper 17 cells	Immunity	Hsin-Jung Wu	1082
7	2014	The microbiome in inflammatory bowel disease: current status and the future ahead	Gastroenterology	Aleksandar D. Kostic	1024
8	2011	Proinflammatory T-cell responses to gut microbiota promote experimental autoimmune encephalomyelitis	Proceedings of the national academy of sciences of the United States of America	Yun Kyung Lee	882
9	2017	Interactions between the microbiota, immune and nervous systems in health and disease	Nature neuroscience	Thomas C. Fung	849
10	2011	Commensal microbiota and myelin autoantigen cooperate to trigger autoimmune demyelination	Nature	Kerstin Berer	808

The number of times used in 180 days reflects the number of times the article has met a user’s information needs, as demonstrated by clicking links to the full-length article on the publisher’s website or by saving the metadata for later use. High usage counts take time to translate into high citations. Still, they have the advantage of novelty, and researchers tend to use newer literature. Still, older literature with higher citations contributes to a secondary increase in its subsequent use [35]. The number of 180-day usages can reflect the current research hot spots and frontiers to a certain extent.

According to Table 3, the first and second most frequently used articles within 180 days were the third and first, respectively, highly cited articles. In third place is an article by Xuan Zhao et al., published in Microbiome in 2022, that disease-resistant phenotypes are related to immunomodulatory function and immune tolerance, with implications for animal husbandry and human health [36]. By analyzing a model of acute colitis developed by Min pigs and Yorkshire pigs, they found that host-microbiota crosstalk contributes to disease resistance phenotypes in three ways: By maintaining part of pattern recognition receptor (PRR) not activated, it can maintain Th2 immune dominance and immune tolerance mode and restore intestinal barrier function to prevent colon diseases [36]. The fourth is the article by Yangxin Li et al. in phytomedicine, where they found that Ershiwuwei Lvxue Pill (ELP), a prescription of Tibetan medicine, can improve joint damage in systemic autoimmune disease rheumatoid arthritis (RA) by inhibiting the production of matrix metalloproteinases (MMPs) and osteoclast activity and regulating intestinal microbiota and host metabolites [37]. The fifth is a review by Liying He et al. published in biomedicine and pharmacotherapy in 2022, in which they summarized the mechanism of interaction between diabetes and gut microbiota and also classified and summarized the natural compounds that treat diabetes through gut microbiota [37]. The sixth on the list, and also on the highly cited list, is a review by Thomas C. Fung, Christina A. Olson, and Elaine Y. Hsiao, published in nature neuroscience in 2017. Microbes influence the activation of peripheral immune cells, which regulate responses to neuroinflammation, brain injury, autoimmunity, and neurogenesis. They discuss the role of CNS resident and peripheral immune pathways in microbiota-gut-brain communication during health and neurological disease [38]. It can be seen that gut microbes impact ADs in multiple body systems.

**Table 3:** Ranking of the top 10 highest 180 days usage

Rank	Year	Title	Journal	First author	Usage count
1	2014	Role of the microbiota in immunity and inflammation	Cell	Yasmine Belkaid	79
2	2009	The gut microbiota shapes intestinal immune responses during health and disease	Nature reviews immunology	June L. Round	66
3	2022	Host-microbiota interaction-mediated resistance to inflammatory bowel disease in pigs	Microbiome	Xuan Zhao	57
4	2022	Ershiwuwei Lvxue Pill alleviates rheumatoid arthritis by different pathways and produces changes in the gut microbiota	Phytomedicine	Yangxin Li	47
5	2022	Regulation of the intestinal flora: a potential mechanism of natural medicines in the treatment of type 2 diabetes Mellitus	Biomedicine and pharmacotherapy	Liying He	46
6	2017	Interactions between the microbiota, immune and nervous systems in health and disease	Nature neuroscience	Thomas C. Fung	40
7	2021	Does the epithelial barrier hypothesis explain the increase in allergy, autoimmunity and other chronic conditions?	Nature reviews immunology	Cezmi A. Akdis	38
8	2022	Metabolite-based dietary supplementation in human type 1 diabetes is associated with microbiota and immune modulation	Microbiome	Kirstine J. Bell	37
9	2021	The gut-joint axis in rheumatoid arthritis	Nature reviews rheumatology	Mario M. Zaiss	35
10	2021	Polysaccharides confer benefits in immune regulation and multiple sclerosis by interacting with gut microbiota	Food research international	Ying Sun	35

## Keywords analysis

### Keywords frequency analysis

**Fig. 3:**
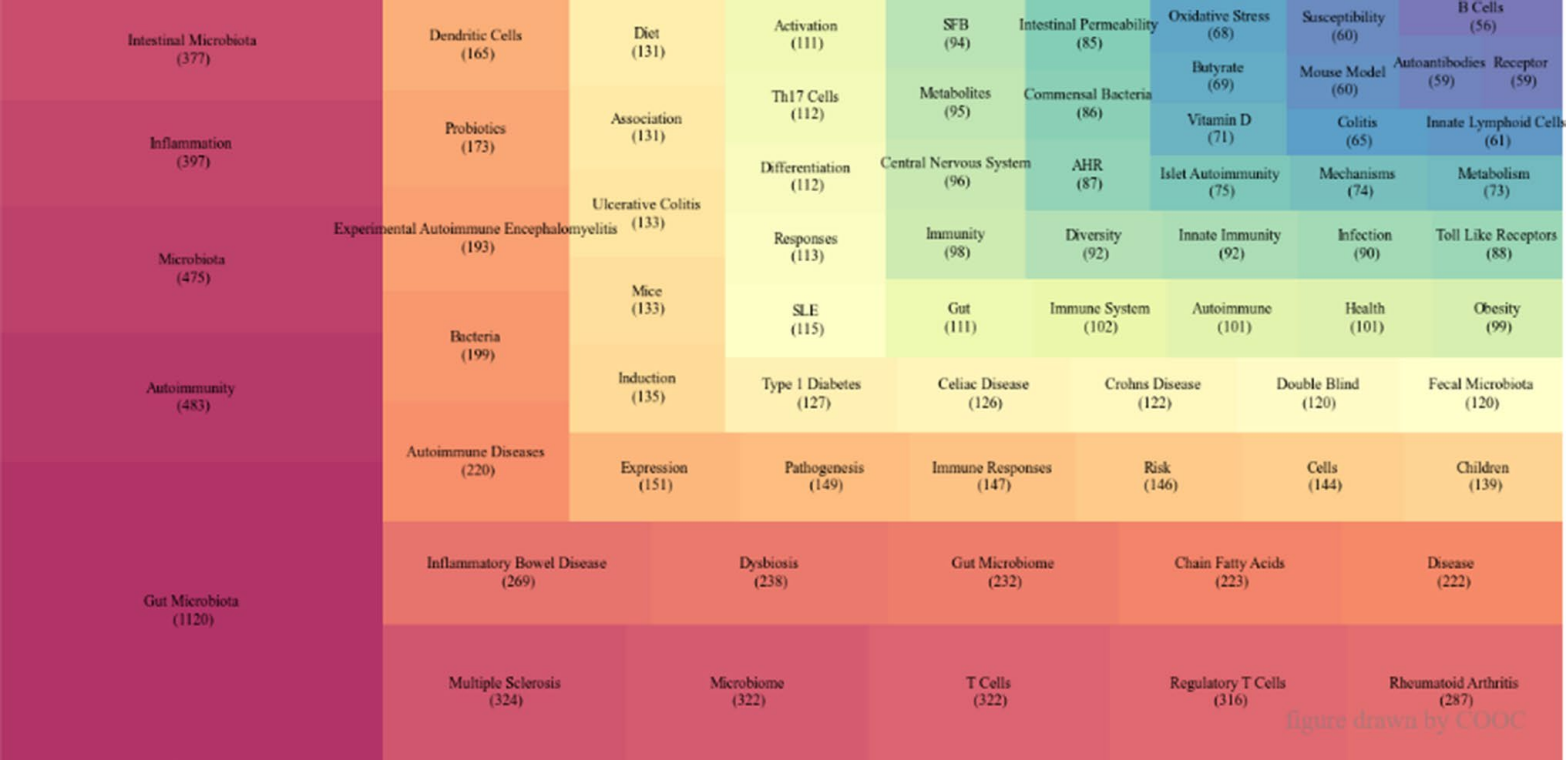
Tree map of top 70 keywords

COOC13.2 was used to extract keywords, and the keywords were synonymously combined. Because keywords overlap in the image, the aryl hydrocarbon receptor, abbreviated as AHR, and the systemic lupus erythematosus abbreviated as SLE. Finally, the top 70 keyword frequencies are reflected in Fig. 3. Keyword frequency is an important index that directly demonstrates the research content, research hot spot, and frontier direction of a field.

## Keywords co-occurrence analysis

**Fig. 4:**
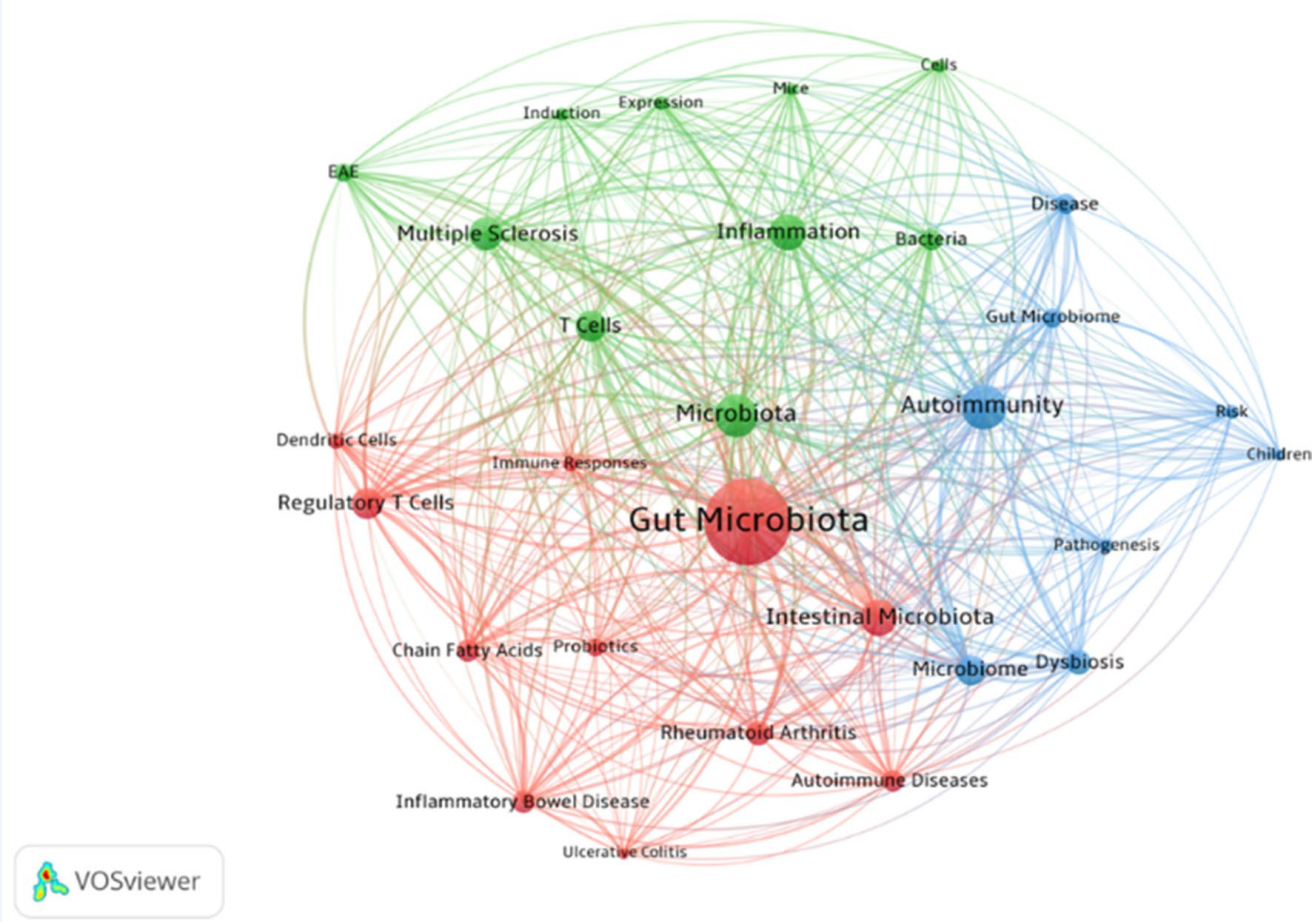
Keywords co-occurrence network

There must be some correlation among the keywords given in the paper, which can be expressed by the co-occurrence frequency. It is generally believed that the more lexical pairs appear in the same literature, the closer the relationship between these two topics will be. Experimental autoimmune encephalomyelitis was abbreviated as EAE because the complete keywords could not be displayed. As shown in Fig. 4, excluding the relationship between the five headings gut microbiota, autoimmunity, microbiota, intestinal microbiota, and microbiome, gut microbiota is associated with inflammation, multiple sclerosis (MS), T Cells, regulatory T Cells (Tregs), RA, inflammatory bowel disease (IBD), and short chain fatty acids (SCFAs). It can be seen that ADs and gut microbiota mainly focus on the immune response, immune cells, and related diseases.

## Keywords, authors analysis

**Fig. 5:**
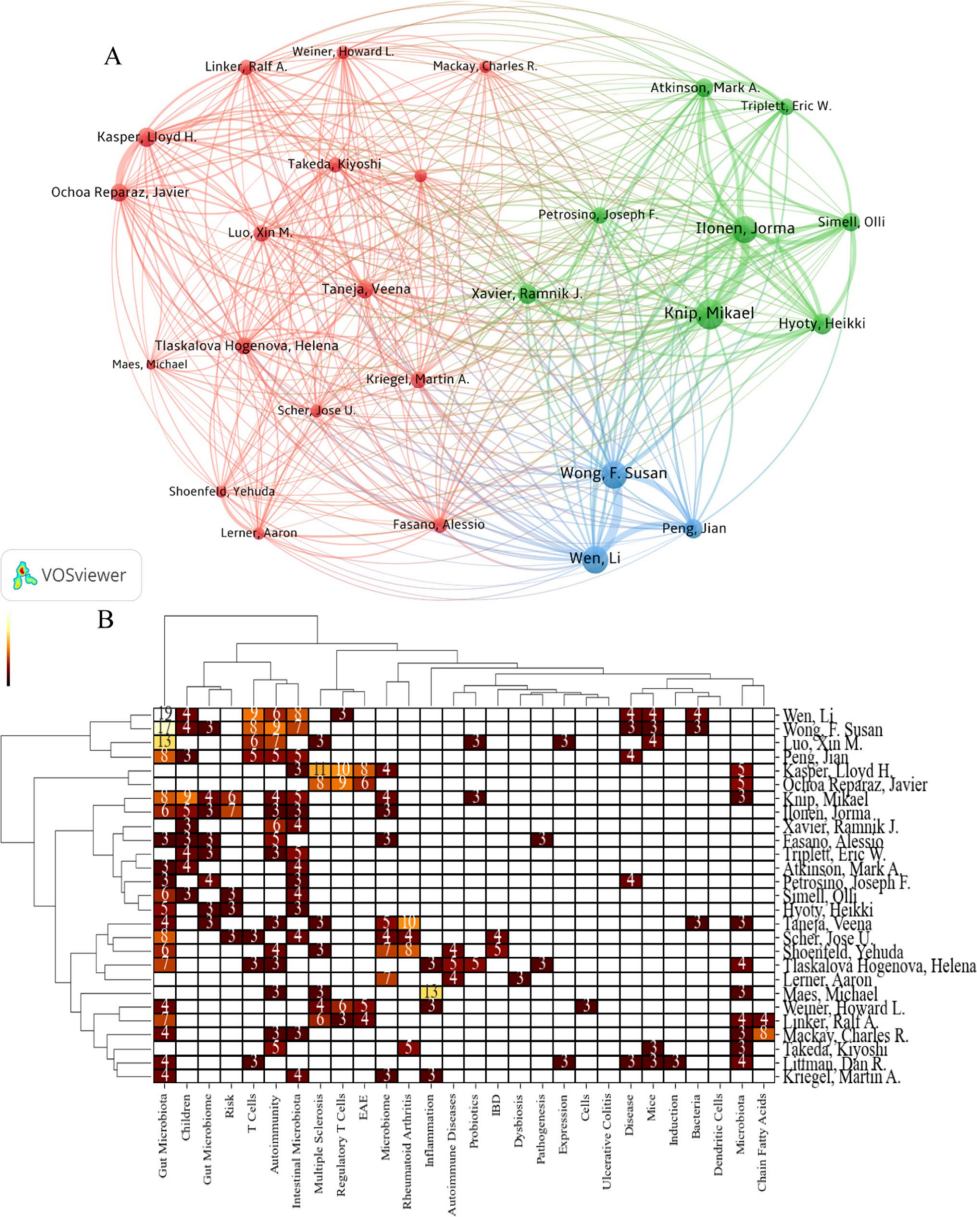
Keywords and authors analysis **A** keywords and authors coupling matrix. **B** Keywords and author two-modular matrix

We use the keyword coupling strength of authors’ works to establish the relationship between authors, draw the corresponding two-mode matrix based on the number of two authors with the exact keywords, and directly display the correlation between authors and keywords in the visualization diagram, which should be able to explicitly and instantly discover the subject knowledge structure centered on the main author and can show the diversity of the author’s academic interests. Compared with the cooperative network of authors in Fig. 2C, Fig. 5A can better reflect the researcher’s research content and seek cooperation between authors in the same research field by coupling keywords with the author. Through Fig. 5A and B can intuitively display the author’s research content. For example, Professor Li Wen has researched gut microbiota, T cells, autoimmunity, intestinal microbiota, Tregs, children, disease, mice, and bacteria. Meanwhile, we can also reflect on the detailed research fields of the authors through Fig. 5A and B, for example, Mikael Knip, Jorma Ilonen, Ramnik J. Xavier, Eric W. Triplett, Mark A. Atkinson, Heikki Hyoty, Olli Simell, and Joseph F. Petrosino, these eight authors have similar research fields.

## Keywords cluster analysis

The high-frequency keywords were analyzed by cluster analysis, and the keywords were classified. According to the co-occurrence color of figure keywords and the understanding of self-related knowledge, the keywords gut microbiota, autoimmunity, microbiota, intestinal microbiota, microbiome, etc., were removed. And they were divided into three categories: A. intestinal regulation, B. immune diseases**,** C. immune-related cells.

## Keywords, time analysis

**Fig. 6:**
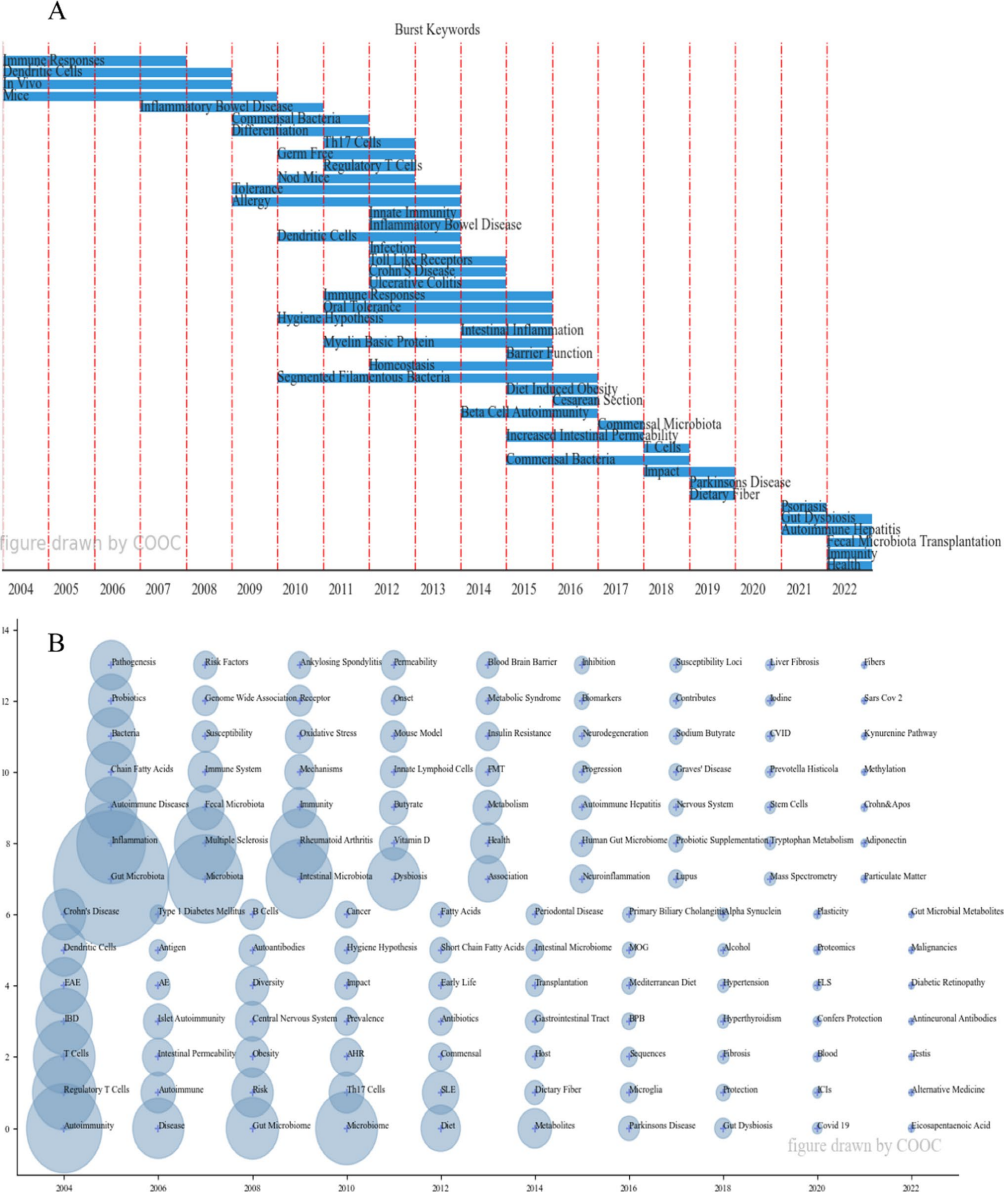
Keywords and time analysis. **A** Burst keywords map. **B** Time zone diagram of theme evolution path

COOC13.2 software is used to draw Fig. 6, which can reflect the changing trend of research topics in the field over time. Figure 6A can focus on the annual keyword mutation, which can better grasp the annual hot issues, and provide a reference for the future research and development of the industry through the mutation words in recent years. By setting the interception frequency of 20 through COOC13.2, Fig. 6A is obtained. Figure 6B. Each circle represents a keyword, and the larger the circle, the higher the frequency of the critical word. The key word is in the year when it first appeared in the analyzed dataset. Once a keyword occurs, it will be fixed to the year it first appeared, although it will still appear in the paper afterward, and will no longer be shown in the figure, only in the year it first appeared. If the keyword appears again in the later years, it will increase the frequency to the position of the keyword for the first time, and the frequency will increase several times. Because keywords overlap in the image, ARS for acute respiratory syndrome, HDP for highly differentiated phenotype, FMT for fecal microbiota transplantation, ICIs for immune checkpoint inhibitors, MOG for myelin oligodendrocyte glycoprotein, BPB for butyrate-producing bacteria, FLS for fibroblast like synoviocytes, and CVID for common variable immunodeficiency. COOC software was used to draw Fig. 6B of topic evolution, reflecting the changing trend of research topics in the field over time.

## Discussion

### General information

The overall number of studies on ADs and gut microbiota showed an upward trend, especially after 2012. The possible reason is that the National Institutes of Health of the United States issued the human microbiome project (HMP) in 2007, and the European Commission announced the launch of the “METAgenomics of the Human Intestinal Tract (MetaHIT)” in 2008. The publication of HMP results in 2012, which might lead to the rapid growth of gut microbiota related research. Afterward, many studies have shown that gut microbiota has a relationship with the immune system, which has also led researchers to focus on ADs with complex etiologies, resulting in the growth of related research on ADs and gut microbiota. From the countries/regions in Table 1, Fig. 2A, the USA (939 articles) has the highest productivity, surpassing second-place China (450 articles). There is close collaboration between countries, especially the USA, with 89 times and 66 times in China and Germany, respectively. In addition, according to Table 1 and Fig. 2B and C, six of the top 10 institutions are from the USA, and the other four were Finland (2), Sweden, and Italy. And seven of the top 10 authors are from the USA, which shows that the related research in the USA is more productive. Furthermore, through Fig. 5, we can know the authors with the same research direction, such as these eight professors, Mikael Knip, Jorma Ilonen, Ramnik J. Xavier, Eric W. Triplett, Mark A. Atkinson, Heikki Hyoty, Olli Simell, and Joseph F. Petrosino, which provides references for cooperation and communication between authors.

### Analysis of research contents and hot spots

The research content can be divided into three categories: A. intestinal regulation**,** B. multisystem ADs, C. immune-associated cells.

Category A includes intestinal regulation, such as dysbiosis, SCFAs, and probiotics. Dysbiosis refers to the disturbance of microbial composition and function associated with mucosal barrier dysfunction and inflammatory response [17, 39] and is associated with ADs, especially RA, type 1 diabetes (T1D), MS, and autoimmune liver disease (AILD), IBD [13, 39]. Dysbiosis can be induced by diet (such as chronic consumption of saturated or trans fats, meat proteins, reducing sugars and salt, and a diet low in fiber [13]), drugs (such as antibiotics [40]), and endogenous factors (such as antimicrobial peptides, S-IgA and mucin layers) [17], mutations in genes (i.e., NOD2 and XBP1) and environmental stress [41]. Therefore, in the relevant studies of dysbiosis in ADs and gut microbiota, the causes and effects of dysbiosis are hot topics. Fatty acids are divided into SCFAs, medium chain fatty acids (MCFAs) and long chain fatty acids (LCFAs). SCFAs are metabolites produced by intestinal microflora through the digestion of complex carbohydrates by fermentation [42]. SCFAs have a variety of mechanisms that inhibit intestinal inflammation and are an inhibitor of histone deacetylase (HDAC), and they can stimulate histone acetyltransferase activity and stabilize hypoxia-inducing factors (HIF) and may induce Tregs either by G-protein-coupled receptors or inhibition of HDAC [42, 43]. LCFAs enhanced the differentiation and proliferation of T helper cell 1 (Th1) and/or T helper cell 17(Th17) cells and damaged their intestinal isolation through the p38-MAPK pathway, which also promoted the autoimmune suppressive T(H)2 immune responses [44, 45]. Its research focuses on treating MS, an autoimmune disease in the nervous system. Probiotics are a common way to regulate gut flora. The main mechanisms of action include enhanced mucosal barrier function, increased abundance of beneficial bacteria, direct antagonism with pathogens, reduced abundance of potentially harmful bacteria in the gut, inhibition of bacterial adhesion and intestinal epithelial invasion, enhanced immune system, and regulation of the central nervous system [46, 47]. In patients with ADs, probiotics can be introduced as a support measure in addition to the standard treatment plan [11]. It has been suggested that the onset of reproductive dysfunction in microbiota can be treated by probiotics using typical species of the genus *Lactobacillus* [48]. Therefore, using probiotics to regulate intestinal flora to treat ADs is a hot spot.

Category B includes MS, RA, IBD, EAE, and ulcerative colitis (UC). MS is a spontaneous immune disease of the nervous system. The microbial-gut-brain axis provides new ideas for treating nervous system diseases such as MS by gut microbiota. The impact of the gut microbiome on immune function through regulation of serotonin production in the gut and complex interactions with immune system components such as T and B cells is important in the development and course of MS [49], and the association of specific gut microbiome with functional changes in MS risk, disease duration and progression, and treatment response [50]. Therefore, the exploration of different intestinal flora provides new ideas for the treatment of MS. Similarly, dietary-microbiome studies have demonstrated that for MS, diet therapy is also one of the directions. As mentioned earlier, SCFAs are one of the dietary modalities used to treat MS. Moreover, identifying gut microbiota-specific IgA cells as systemic mediators of MS has broad implications as a useful biomarker and IgA-producing cells as an immune subset for therapeutic intervention [51]. And, EAE is an animal model of MS [52]. For animal models, the main role is to simulate the effects of various experiments on MS and explore the mechanism of MS, including intestinal flora. For RA, which is a chronic autoimmune inflammatory disease that primarily affects joints, gut microbiota and/or gut barrier function may help prevent or treat RA [53]. DMARDs that regulate the gut-joint axis can change the composition of gut microbiota [53], and drugs that regulate gut microbiota or anti-inflammatory drugs that require intestinal activation also play a role in the development of drugs for the treatment of RA [54]. Additionally, diets such as acarbose, probiotics, and prebiotics play a role in the prevention of RA [55]. IBD, including Crohn’s disease (CD) and UC, is a multifactorial chronic disease of the gastrointestinal tract [56]. Specific classes of metabolites of gut microbes, especially bile acids, SCFAs, and tryptophan metabolites, have been implicated in the pathogenesis of IBD [57]. In terms of diet, there have been attempts to treat IBD by modulating the gut microbiome with probiotics, prebiotics, antibiotics, FMT, and genetic manipulation [58]. And several cross-sectional reports suggest that a gluten-free diet (GFD) may improve symptoms in patients with IBD [59]. For CD, the bacterial-metabolite interaction network of sulfur metabolism is a key mechanism associated with CD activity [60]. Nutritional strategies for children with CD are exclusive enteral nutrition (EEN), partial enteral nutrition (PEN), Crohn’s disease elimination diet (CDED), and Crohn’s disease with diet therapy (CD-Treat) [61]. For UC, the treatment of UC by specific strains is one of the directions. For example, engineered *S. cerevisiae* can be used as an effective and safe treatment strategy for UC by inhibiting macrophage pyroptosis and regulating intestinal microbiota [62]. And substances that affect gut microbiota have been isolated from medicinal herbs (e.g., SP2-1, from *Scutellaria baicalensis* georgi [63], rhein, from rhubarb [64], evodiamine, from evodia fructus [65]) to treat UC. Furthermore, FMT by colonoscopic infusion or enema or by oral administration may all be promising and feasible treatment options for UC [66], and multidonor FMT with an anti-inflammatory diet can effectively induce deep remission of mild-moderate UC for more than one year [67]. Moreover, T1D, celiac disease (CeD), CD, and SLE, although they did not appear in the keyword co-occurrence network (30 keywords), Fig. 3 shows that they ranked 32, 33, 34 and 37, respectively, in the list of high-frequency keywords, and there are many related studies. These are also key concerns in gut microbiota and ADs. Among them, T1D and CeD are also closely related to pediatric research [68–71], and CD belongs to IBD mentioned above.

Category C includes T cells, Tregs, and dendritic cells. T cells are closely related to intestinal microbes and ADs, affect the stability of intestinal microbes, and are also regulated by intestinal microbes [72]. Treg is a kind of T cells, which has clinical potential as a cell therapy for the treatment of autoimmunity [73]. The immune imbalance between anti-inflammatory Tregs and proinflammatory Th17 is associated with a variety of ADs [74, 75]. Attention has been paid to the influence of intestinal microbiota [74], and Tregs have also been associated with intestinal dysbiosis [76]. Tregs, as mentioned above, are regulated by SCFAs and LCFAs, and some altered commensal communities can enhance the mitochondrial fitness of intestinal Tregs [77]. Th17 cells are also a type of T cells, which also play an important role in ADs and gut microbiome research [72, 78], ranking 39 in the high-frequency keywords. Dendritic cells, one of the major professional antigen-presenting cells, are also affected by gut microbiota-derived metabolites [79], such as SCFAs [80], taurine deoxycholic acid (TCDCA) [81], and secondary bile acids (BAs)[82]. Among them, BAs regulate DC mainly through TGR5 [81, 82].

### Frontier analysis

According to Fig. 6A, burst keywords can find annual hot issues. In 2012, when the data began to grow rapidly, we can see that the most prominent keywords are Th17 Cells (2011–2012), Germ Free (2010–2012), Tregs (2011–2012), Nod Mice (2010–2012), Tolerance (2009–2013), Allergy (2009–2013), dendritic cells (2010–2013), etc. As for 2021 and 2022, the most prominent keywords are psoriasis (2021), gut dysbiosis (2021–2022), AIH (2021–2022), and FMT (2022).

Psoriasis is an immune-mediated systemic disease that affects approximately 125 million people worldwide with profound skin and intestinal dysbiosis [83–85]. Tregs deficiency contributes to the pathogenesis of psoriasis and may be attributed to enhanced suppression and/or impaired stimulation of Tregs [86]. Oral probiotics, prebiotics, and fecal microbial transplantation are most evident in providing health benefits for patients with psoriasis [84, 87]. In addition, *n*-3 polyunsaturated fatty acids, vitamin D, vitamin B12, SCFAs, selenium, genistein, and dietary fibers are also beneficial for psoriasis, and deficiencies in vitamin D or selenium have also been associated with intestinal disorders [83]. As mentioned above, gut dysbiosis is related to a variety of ADs [13, 39]. Gut dysbiosis is not only a hot spot but also a frontier in ADs and gut microbiota.

AIH is a chronic immune-mediated liver disease that is distributed in all ethnic groups worldwide with increasing prevalence [88]. The gut microbiota can be used as a non-invasive biomarker to assess the potential of autoimmune hepatitis [89]. Probiotics and FMT, which may be involved in the regulation of the immune imbalance of follicular regulatory T(TFR) and helper T(TFH) cells and the recovery of IM composition, as well as targeting signaling pathways associated with the gut microbiome, which has provided new insights into the treatment of patients with AIH [88, 90]. The emergence of AIH also reflects the in-depth and related research on the gut-liver axis/liver-microbiome axis and AIH to a certain extent. In addition to AIH, AILD also contains primary biliary cholangitis (PBC) and primary sclerosing cholangitis (PSC) [91]. For PBC and PSC, they may also become the frontier of ADs and gut microbiota like AIH.

FMT was mentioned above as a potentially promising and feasible treatment option for the treatment of UC [66]. For psoriasis, FMT is one of the most effective modalities [84], as well as one of the treatment modalities for AIH [88, 90]. It is also effective against systemic sclerosis and T1D [92]. FMT can be optimized as a tailored dietary intervention pair, facilitating a pathway for precise engineering of the gut microbiome using the diet in ADs [93]. It can be seen that FMT is effective and relatively safe in the treatment of ADs and is expected to be used as a method to induce remission of active ADs [92]. Thus, from Fig. 6A, we think that psoriasis, gut dysbiosis, AILD, and FMT may be the future research direction.

### Strength and limitation

This study provides the first intuitive, objective, accurate, and comprehensive systematic analysis of ADs and gut microbiota publications and their trends, which can provide comprehensive guidance for clinicians and scholars in the field. Literature metrology and visual analysis can help researchers intuitively understand the research hot spot, evolution, and development trend of ADs and gut microbiota. Inevitably, there are some limitations to the study. First, the literature included in our study may not be exhaustive. For one thing, our study only examined data from the Web of Science SCI-E database. Therefore, the articles identified may not adequately reflect all ADs and gut microbiota studies, and more detailed studies are expected in the future.

## Conclusion

In conclusion, from the annual publication volume of related literature, gut microbiota has attracted more and more attention to ADs. Europe and the USA have made the greatest contribution to this field, and the cooperation between them is closer, and the publications are more concentrated. The research focus is mainly on dysbiosis, SCFAs, and probiotics for the regulation of gut microbiota, the impact of gut microbiota on MS, RA, IBD, EAE, UC, as well as T1D, CeD, CD, SLE, and related immune cells involved in ADs and gut microbiota, such as T cells, Tregs, Dendritic cells, as well as Th17. As for the ADs and gut microbiota research frontier, it is possible to focus on gut dysbiosis, which is also involved in hot spots. In addition, psoriasis, AILD (including AIH, PBC, PSC), and FMT may also be future research directions. These findings can help clinicians and researchers understand ADs and gut microbiota research hot spots and provide references for future research directions.

## Data Availability

The datasets generated during the current study are available in the Web of Science (http://www.webofknowledge.com).
